# Elevated blood lactate in resting conditions correlate with post-exertional malaise severity in patients with Myalgic encephalomyelitis/Chronic fatigue syndrome

**DOI:** 10.1038/s41598-019-55473-4

**Published:** 2019-12-11

**Authors:** Alaa Ghali, Carole Lacout, Maria Ghali, Aline Gury, Anne-Berengere Beucher, Pierre Lozac’h, Christian Lavigne, Geoffrey Urbanski

**Affiliations:** 1 Department of internal medicine, University Hospital, Angers, 49000 France; 2 Department of general medicine, Faculty of medicine, Angers, 49000 France

**Keywords:** Neurology, Biological techniques

## Abstract

Elevated blood lactate after moderate exercise was reported in some of patients with myalgic encephalomyelitis/chronic fatigue syndrome (ME/CFS). We hypothesised that blood lactate could be also elevated in resting conditions. We aimed investigating the frequency of elevated lactate at rest in ME/CFS patients, and comparing characteristics of ME/CFS patients with and without elevated lactate. Patients fulfilling international consensus criteria for ME/CFS who attended the internal medicine department of University hospital Angers-France between October 2011 and December 2017 were included retrospectively. All patients were systematically hospitalised for an aetiological workup and overall assessment. We reviewed their medical records for data related to the assessment: clinical characteristics, comorbidities, fatigue features, post-exertional malaise (PEM) severity, and results of 8 lactate measurements at rest. Patients having ≥1 lactate measurement ≥2 mmol/L defined elevated lactate group. The study included 123 patients. Elevated (n = 55; 44.7%) and normal (n = 68; 55.3%) lactate groups were comparable except for PEM, which was more severe in the elevated lactate group after adjusting for age at disease onset, sex, and comorbidities (OR 2.47, 95% CI: 1.10–5.55). ME/CFS patients with elevated blood lactate at rest may be at higher risk for more severe PEM. This finding may be of interest in ME/CFS management.

## Introduction

Myalgic encephalomyelitis also known as chronic fatigue syndrome (ME/CFS) is a long-term and debilitating multisystem condition of unknown aetiology affecting several millions of individuals worldwide^1,2^. It represents a significant public health issue due to the high levels of health care resource use by ME/CFS patients, and the resulting loss of productivity^3^. ME/CFS is characterised by persistent or relapsing unexplained fatigue of at least 6 months duration that is not alleviated by rest and leads to a substantial reduction of previous levels of different activities of patients. Beside fatigue, ME/CFS patients report many symptoms, especially unrefreshing sleep, cognitive difficulties, orthostatic intolerance, and pain. The frequency and the severity of symptoms differ greatly between patients and over the course of the disease. Nevertheless, the cardinal feature of ME/CFS is the post-exertional malaise (PEM), which is defined as the exacerbation of some or all of a patient’s symptoms due to physical or cognitive stressors that were normally tolerated before disease onset^4^. PEM helps distinguish ME/CFS from other causes of chronic fatigue such as depression and fibromyalgia (FM), and recent diagnostic criteria of ME/CFS require its presence^2,4^.

At present the underlying mechanism of ME/CFS is not yet well defined and its diagnosis, in the absence of a specific diagnostic marker, is based on recognised criteria^2,4,5^, and established after eliminating other diseases that could contribute to the patient’s complaints.

Giving to the fact that ME/CFS is a heterogeneous illness, many studies tried to subtype ME/SFC patients according to various clinical variables such as number of symptoms^6^, type of fatigue^7^, mode of illness onset^8^, age at onset^9^, and illness course. Biological variables were also used to group ME/CFS patients including natural killer cell activity^10^, cytokines^11^, and gene expression in the peripheral blood^12^.

Mitochondrial dysfunction is known to be linked to fatigue^13^, and almost all studies that investigated mitochondrial function in ME/CFS patients observed dysfunction, for instance low levels of carnitine^14^, and CoQ10^15^, and elevated ventricular lactates^16,17^. During exercise, low levels of ATP^18^ and elevated blood lactate concentration^19^ were found in some ME/CFS patients. High levels of oxidative stress markers were observed not only after exercise^20^ but also at rest^21^ and correlated with CFS symptoms^22^.

We hypothesised that a proportion of ME/CFS patients could display abnormal blood lactate concentrations at resting conditions as a result of mitochondrial dysfunction. We thus aimed to examine whether this group of patients represent a distinct phenotype.

## Results

**Figure 1:**
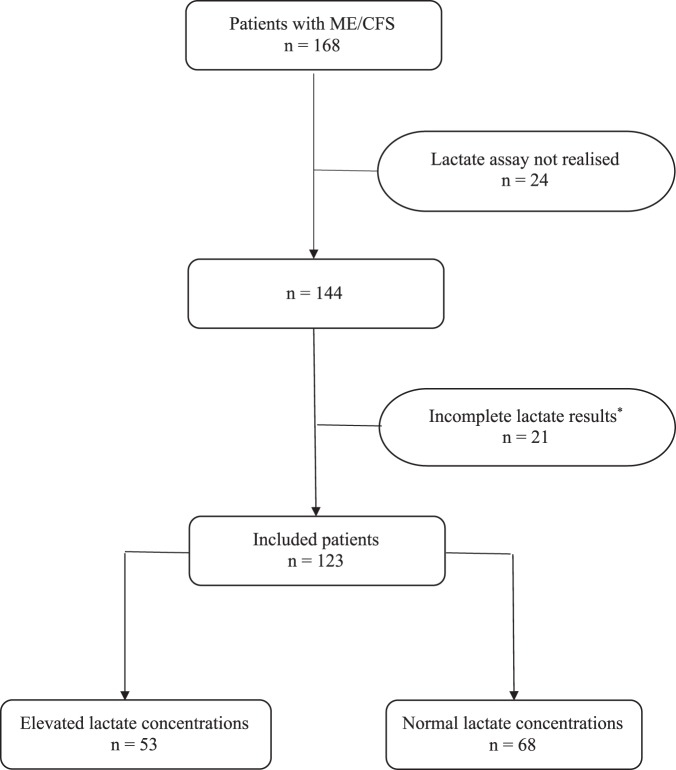
Flow chart of patients’ inclusion. Notes: ME/CFS: Myalgic encephalomyelitis/Chronic fatigue syndrome. ^*^More than 2 out of 8 time points measurements are missing due to haemolysis.

Among the 168 patients who were fulfilling inclusion criteria, we excluded 45 patients in whom 24 did not undergo lactate assessment, and 21 patients in whom results of lactate assay were incomplete, mainly due to blood sample haemolysis. Hence a total of 123 patients constituted the study population (Fig. 1).

**Figure 2:**
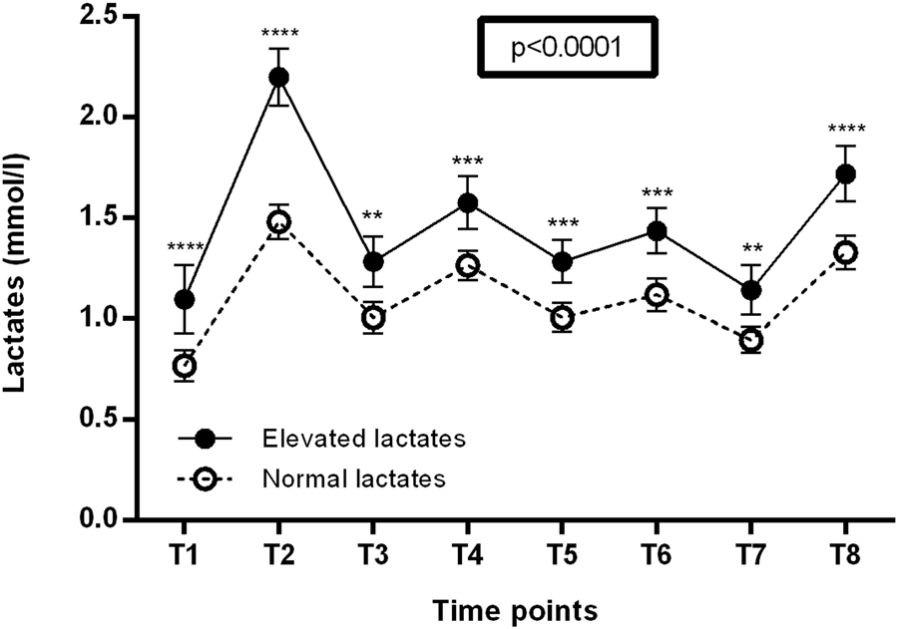
Comparison between the overall blood lactate levels in the elevated lactate group and the normal lactate group. Notes: The solid line represents the curve of blood lactate of the elevated lactate group, and the dotted line represents the curve of blood lactate of the normal lactate group. Blood lactate concentrations were measured for each patient at 8 time points on a one-day period: T1 = before breakfast after overnight fasting for 16 hours; T3, T5, and T7 = 30 minutes before lunch, 4 pm snack, and dinner at 4-hour intervals; T2, T4, T6, T8 = 1 hour after the 4 meals. The comparison of both curves was realized by a 2-way ANOVA, and the numerical p-value on the graph is tied to the interaction factor. Lactate values are presented as means with 95% confidence interval. Post-hoc analyses for each lactate time point were realised with Bonferroni corrected t-tests and p-values of these tests were summarised as following: **p < 0.01, ***p < 0.001 and ****p < 0.0001.

Table 1 presents the characteristics of the study population. The PEM assessment showed that the median PEM score on the CDC SI questionnaire was equal to 12 [7.5–12]. A more severe PEM (scores ≥ 12) were observed in 62 (50.4%) patients. Elevated blood lactate levels at rest ≥2 mmol/L on one or more measurements were observed in 55 (44.7%) patients, who constituted the elevated lactate group. Data concerning all lactate measurements can be found as Supplementary Table S1. Figure 2 shows that the overall lactate levels in the elevated lactate group were significantly higher (p < 0.0001) than those of the normal lactate group. Lactate values significantly differed between the 2 groups for each time point.

**Table 1:** Demographic characteristics, fatigue features, clinical manifestations, comorbid conditions, and laboratory abnormalities of the study population.

Epidemiological characteristics	
Patients, n (%)	123
Female/Male	89/34
Duration of illness (months)	72 [36–120]
Age at disease onset (years)	34 [26–41]
13–19 n (%)	11 (8.9)
20–29 n (%)	34 (27.6)
30–39 n (%)	42 (34.1)
40–49 n (%)	23 (18.7)
≥50 n (%)	13 (10.6)
Family history of fatigue	13 (10.6)
Identified disease trigger	87 (70.7)
Infectious triggers	58 (47.2)
**Fatigue features**	
Fatigue severity scale (n = 96)	5.5 [5–6.2]
Fatigue scale (n = 103)	24 [20–27.5]
Modified fatigue impact scale (n = 104)	
Physical subscale	29 [25–32]
Cognitive subscale	27 [20–32]
Psychosocial subscale	6 [4–6.5]
**Clinical manifestations, n (%)**	
More severe post-exertional malaise*	62 (50.4)
Difficulty processing information	118 (95.9)
Short-term memory loss	101 (82.1)
Headaches	89 (72.4)
Myalgia	107 (87)
Arthralgia	72 (58.5)
Disturbed sleep patterns	109 (88.6)
Unrefreshed sleep	118 (95.9)
Neurosensory and perceptual disturbances	114 (92.7)
Motor disturbances	112 (91.1)
Flu-like symptoms	92 (74.8)
Recurrent infections	47 (38.2)
Gastrointestinal impairments	105 (85.4)
Urinary impairments	38 (30.9)
Orthostatic intolerance	41 (31.3)
Palpitation	79 (64.2)
**Main comorbidities, n (%)**	
Reactive depression	36 (29.3)
Fibromyalgia	19 (15.5)
Irritable bowel syndrome	50 (40.7)
**Main laboratory abnormalities, n (%)**	
Elevated blood lactate at rest^†^	55 (44.7%)
Serum zinc deficit^‡^	39 (31.7)
Serum 25-hydroxyvitamin D deficit^§^	61 (49.6)
Low plasma 8 am and/or 8 pm cortisol levels^¶^	23 (18.7)

Notes: ^*^Score ≥12 on Centres for Disease Control and Prevention Symptom Inventory auto-questionnaire. ^†^One or more blood lactate measurements at rest. ^‡^Normal range = 0.70–1.25 mg/L, measured by atomic emission spectroscopy/high-frequency induction plasma. ^§^Normal range = 75–250 nmol/L, measured by chemiluminescence technology (CLIA). ^¶^Normal values = 5–49 µg/L at 8 am, and 30–100 µg/L at 8 pm, measured by Immunoenzymatic chemoluminescznce (12000 ABBOT) method. Cortisol deficit was retained if one or both measurements were reduced. Categorical data were expressed as absolute number and percentage. Continuous data were expressed as median and interquartiles.

As summarised in Table 2, the comparison between the group of patients with elevated lactate levels and the group of those with normal lactate showed that the only significant difference between both groups was the number of patients who display more severe PEM, which was significantly higher in the elevated lactate group (34/55; 61.8%) than in the normal lactate group (28/68; 41.2%), (p = 0.02). This difference persisted after adjusting for age at disease onset, sex, comorbidities, and laboratory findings with an OR for more severe PEM in the elevated lactate group of 2.47 [95% CI: 1.10–5.55] (p = 0.03) (Table 3).

**Table 2:** Comparison of patients according to blood lactate levels at rest.

	Elevated lactate	Normal lactate	p
**Epidemiological characteristics**			
Patients, n (%)	55 (44.7)	68 (55.3)	
Female/Male	35/20	54/14	0.052
Duration of illness (months)	60 [63–96]	78 [36–144]	0.11
Age at disease onset (years)	36 [25.5–42]	32 (26–39]	0.26
**Fatigue features**			
Fatigue severity scale	(n = 43) 5.6 [4.8–6.2]	(n = 53) 5.4 [5.1–6]	0.90
Fatigue scale	(n = 46) 24 [21–28]	(n = 57) 23 [17–27]	0.14
Modified fatigue impact scale Physical subscale Cognitive subscale Psychosocial subscale	(n = 47) 28 [24–32] 28 [20–33] 6 [4–7]	(n = 57) 30 [27–32] 26 [20–32] 6 [4–6]	0.09 0.59 0.43
**Patients’ comorbidities, n (%)**			
Reactive depression	15 (27.3)	21 (30.9)	0.66
Fibromyalgia	8 (14.6)	11 (16.2)	0.80
Irritable bowel syndrome	18 (32.7)	32 (47.1)	0.11
**Clinical manifestations n (%)**			
More severe post-exertional malaise^*^	34 (61.8)	28 (41.2)	**0.02**
Difficulty processing information	54 (98.2)	64 (94.1)	0.26
Short-term memory loss	46 (83.6)	55 (80.9)	0.69
Headaches	42 (76.4)	47 (69.1)	0.37
Myalgia	50 (90.9)	57 (83.8)	0.25
Arthralgia	36 (65.5)	36 (53)	0.16
Disturbed sleep patterns	51 (92.7)	58 (85.3)	0.20
Unrefreshed sleep	51 (92.7)	67 (98.5)	0.11
Neurosensory and perceptual disturbances	48 (87.3)	66 (97.1)	0.16
Motor disturbances	50 (90.9)	61 (89.7)	0.56
Flu-like symptoms	42 (76.4)	50 (73.5)	0.72
Recurrent infections	25 (45.5)	22 (32.4)	0.14
Gastrointestinal impairments	43 (78.2)	62 (91.2)	0.63
Urinary impairments	15 (27.3)	23 (33.8)	0.43
Orthostatic intolerance	15 (27.3)	26 (38.2)	0.20
Palpitation	35 (61.8)	44 (64.7)	0.90
Laboratory abnormalities, n (%)			
Serum zinc deficit^†^	20 (36.4)	19 (28)	0.31
Serum 25-hydroxyvitamin D deficit^‡^	31 (56.4)	30 (44.1)	0.18
Low plasma 8 am and/or 8 pm cortisol levels^§^	10 (18.2)	13 (19.1)	0.90

Notes: ^*^Score ≥12 on Centres for Disease Control and Prevention Symptom Inventory auto-questionnaire. ^†^Normal range = 0.70–1.25 mg/L, measured by atomic emission spectroscopy/high-frequency induction plasma. ^‡^Normal range = 75–250 nmol/L, measured by chemiluminescence technology (CLIA). ^§^Normal values = 5–49 µg/L at 8 am, and 30–100 µg/L at 8 pm, measured by Immunoenzymatic chemoluminescznce (12000 ABBOT) method. Cortisol deficit was retained if one or both measurements were reduced. Categorical data were expressed as absolute number and percentage. Continuous data were expressed as median and interquartiles.

**Table 3:** Multivariate analysis of severe post-exertional malaise in the group of elevated lactate.

	p-value	OR [95% CI]
Sex (male)	0.42	1.46 [0.58–3.66]
Age at onset disease ≥34 years	0.93	1.04 [0.46–2.31]
PEM score ≥12	0.03	2.47 [1.10–5.55]
Reactive depression	0.81	0.86 [0.37–2.17]
Fibromyalgia	0.52	0.570[0.23–2.12]
Irritable bowel syndrome	0.31	0.66 [0.29–1.48]
Serum zinc deficit	0.44	1.41 [0.59–3.35]
Serum 25-hydroxyvitamin D deficit	0.38	1.45 [0.63–3.33]
Low plasma cortisol levels	0.77	1.16 [0.43–3.17]

Notes: OR [95% CI]: Odds Ratio with 95% Confidence interval; PEM: post-exertional malaise; Multivariate analysis was performed with logistic regression. The variable to explain was the lactate group (elevated or normal). The variables included in the model were those present in the table as well treated sleep apnoea syndrome (p = 0.10) and treated Hashimoto’s thyroiditis (p = 0.41).

## Discussion

ME/CFS is characterised by persistent or recurrent fatigue and the occurrence of PEM, which is a key feature of this debilitating illness. Due to the heterogeneous character of ME/CFS and the absence of biological marker, many studies tried to subtype ME/CFS patients on the basis of clinical or biological variables. Mitochondrial dysfunction has been shown in some of ME/CFS patients^14–22^. In the current study we identified a group of ME/CFS patients with elevated blood lactate in resting conditions and displaying more severe PEM.

The demographic characteristics, clinical manifestations, and comorbid conditions of our population were comparable to that reported in prior studies. For instance, the age peak in the age range 30–39 (34% of patients) and the median age of disease onset of 34 [26–41] years^23^, the higher prevalence of women and the median duration of illness^1^, and the family history of chronic fatigue syndrome (CFS) in one or more first-degree relatives (11% of patients)^24^ were in accord with that previously reported. Nevertheless, the percentage of patients who identified at least one ME/CFS precipitating factor (70.7%) and that of those who reported an infectious trigger (47.2%) was somewhat lower than that of prior studies^24,25^.

One or more comorbidities were found in 76 (61.8%) patients, which corresponds to previous reports that found comorbidities prevalence between 50 and 65%^26,27^ but higher prevalence was reported in one study^28^.

Laboratory anomalies observed in our population including reduced 8am and/or 8 pm plasma cortisol levels (18.7%), low serum zinc (32%), and low serum 25-hydroxy vitamin D (49.6%) were comparable to that reported in literature^29–31^.

Blood lactate assay results showed elevated lactate levels in resting conditions in 55 (44.7%) patients. Lane *et al*. found elevated lactate concentrations in a proportion of CFS patients (31/96; 32%) after short periods of exercise below the predicted anaerobic threshold^19^. They also reported that patients displaying abnormal lactate responses to exercise had relative deficiency of mitochondria type I fibres on muscle biopsies, which may explain elevated lactate responses to exercise^32^, and low intracellular pH during recovery phase when examined by magnetic resonance spectroscopy of muscle^33^. Other teams showed elevated ventricular lactates in CFS patients pointing to metabolic dysfunction in these patients^16,17^. In the current study, almost half of patients had elevated blood lactate concentrations in resting conditions. To the best of our knowledge, our study is the first to show these results. Lactate values were significantly different between the 2 groups and this difference was observed for each time point. Nevertheless, this difference appeared to be more prominent at T2. A hypothesis that could explain, at least in part, the predominant elevation of lactate levels at T2 is that eating after 16-hour fasting would abolish the beneficial effect of fasting on mitochondrial functioning and redox signalling^34^, leading to a sort of rebound effect responsible for mitochondrial dysfunction with increased blood lactate concentration after breakfast. The positive effect of fasting was reported in FM patients^35^, which could be interesting to evaluate its impact in ME/CFS patients.

When we classified our study population into two groups with or without elevated lactate to compare their phenotypic characteristics, we found that they only differ in the number of patients experiencing more severe PEM, which was significantly higher in patients with elevated lactate. This association persisted after adjusting for age at disease onset, sex, and comorbidities (OR 2.47, 95% CI: 1.10–5.55; p = 0.03). PEM severity was assessed in all patients by the standardised CDC SI self-reported questionnaire^36^, which includes an item designed to measure the presence, frequency, and intensity of PEM. The CDC SI is one of two tools proposed by the IOM^2^ for PEM assessment. We used the median PEM score on the CDC SI questionnaire to set the cut-off score ≥12 defining more severe PEM in our study.

Elevated blood lactate in resting conditions in ME/CFS patients was not reported before. In our study, patients did not display a state of extreme fatigue at the time of lactate assay, and strict resting conditions were followed carefully in all patients all over the period of different samples’ collection during hospitalisation. As well, all patients received standardised meals. Thus, the rise of lactate levels in a proportion of patients could not be related to exertion or due to variation in caloric intake.

It is interesting to note that although the whole study population was suffering from high levels of fatigue and fatigue-related impairment, results of fatigue scales showed that fatigue severity was comparable between both groups; FS median score (elevated lactate group 24 [21–28] vs. normal lactate group 23 [17–27]; p = 0.14) and FSS median score = (elevated lactate group 5.6 [4.8–6.2] vs. normal lactate group 5.4 [5.1–6]; p = 0.90). In this way, elevated lactate levels were correlated with PEM severity, but not with fatigue severity. One can expect that patients who display severe PEM are suffering from high level of general fatigue, however some authors report that some of patients who experienced severe PEM did not report high fatigue levels because they were reducing their activities to stay within their energy envelope and so their fatigue levels may be low but they can experience exhaustion if they exceed their functional capacities or exposed to a PEM trigger^37,38^. Another possible explanation is that there is currently no gold standard measurement of fatigue, and a ceiling effect for FSS and FS was reported^39^ so these scales would not accurately reflect fatigue severity.

### Study’s limitations

Although data of our study were collected retrospectively, all patients were examined and diagnosed by the same physician, and all patients underwent a same standardised procedure in terms of clinical assessment including PEM, and laboratory investigations, especially lactate assay.

One source of weakness in this study was the lack of data concerning fatigue assessment as well its impact on function in some of our patients. However, these data were not statistically different in the 2 groups. As well, data concerning mode of onset of the disease were unavailable for a great number of patients and thus unexploitable. Finally, the MFIS scale that we used to measure the impact of the fatigue on function has been mainly validated in multiple sclerosis patients, but not in those with ME/CFS. Nevertheless, we have used this scale alongside two other scales, FS and FSS, which explore fatigue severity as recommended by the CDC-NINDS project^40^.

At the best of our knowledge, this is the first study that reports elevated blood lactate in resting conditions in a significant proportion of patients with ME/CFS. Patients who showed abnormal elevation of blood lactate at rest displayed more frequent severe PEM than those with normal lactate concentrations. This finding brings supplementary evidence for mitochondrial dysfunction in ME/CFS patients, and may contribute to a better understanding the illness. Subtyping ME/CFS patients adds to the growing body of evidence that ME/CFS is heterogeneous, and allows identifying patients with more risk for severe PEM who must adhere more closely to pacing strategies in order to avoid PEM occurrence and prevent disease exacerbation. Furthermore, our study allowed describing clinical and biological characteristics of a French population with MPE/CFS. Shedding light on these characteristics may improve knowledge and raise awareness of this public health issue.

## Methods

### Ethics

The study was approved by ethics committee of the University hospital of Angers (2018/44) and was conducted in compliance with the Helsinki agreement. Data collection was approved by the French institutional authority for data protection (CNIL).

### Study population and data collection

We reviewed all medical records of patients attending the outpatient clinic of the internal medicine department of Angers University Hospital and diagnosed as having ME/CFS between October 1, 2011 and December 31, 2017. The diagnosis of ME/CFS was established by the same physician after a systematic 3-day hospitalisation during which an aetiological workup and an overall assessment of fatigue features and PEM severity were realised for each patient. We enrolled retrospectively all adult patients aged ≥18 years who met the International Consensus Criteria (ICC) 2011^4^. In accordance with these criteria, patients with an identifiable medical condition that could account for chronic fatigue, and those with primary psychiatric disorders or substance dependence were excluded. Patients with diseases or drugs susceptible to be associated with elevated lactate levels were also excluded. According to ICC 2011, comorbidities such as FM, irritable bowel syndrome, Hashimoto’s thyroiditis, and reactive depression did not constitute an exclusionary condition.

Epidemiological characteristics, fatigue features, and comorbidities were extracted from patients’ medical records as well as reported patients’ symptoms including the frequency and intensity of PEM.

The following biological data were also gathered for each patient: lactate assessment, immunological assay, infectious disease screen for HIV, hepatitis B and C, Lyme disease, enterovirus, *Mycoplasma pneumoniae, Chlamydia pneumoniae*, serum zinc, 25- hydroxyvitamin D, morning (8 am) and evening (8 pm) plasma cortisol, testosterone, iron studies, plasma vitamin B12 and serum folate, full blood count and differential, erythrocyte sedimentation rate, electrolytes, calcium, phosphate, fasting glucose, C-reactive protein, liver function, serum protein electrophoresis, renal function, thyroid function, and creatine kinase.

### Fatigue assessment tools

Fatigue level was assessed by means of standardised auto-questionnaires; the fatigue scale (FS)^41^ and the fatigue severity scale (FSS)^42^. The impact of fatigue on patient’s activities was assessed by the modified fatigue impact scale (MFIS)^43^.

### PEM assessment

The PEM item from the standardised auto-questionnaire of Centres for Disease Control and Prevention Symptom Inventory (CDC SI)^36^ was used to measure PEM symptoms over the past month. Perceived frequency of PEM was rated on a 4-point scale (1 = a little of the time, 2 = some of the time, 3 = most of time, 4 = all of the time), and its intensity was measured on a 3-point scale (1 = mild, 2 = moderate, 3 = severe). The intensity score was converted into equidistant score (0 = symptom not reported, 1 = mild, 2.5 = moderate, 4 = severe). The frequency and intensity scores were then multiplied to create the PEM severity score ranging from 0–16. In the absence of validated threshold that defines PEM severity, we used the median PEM score on the CDC SI questionnaire to set the cut-off score ≥12 defining more severe PEM.

### Lactate assay

The following procedure was applied for all patients included in the study: each patient was admitted at 3 pm (day 1). A peripheral venous catheter was inserted as soon possible after admission and patient was asked to follow resting and fasting conditions (drinking water was allowed till midnight) until next day (day 2). At day 2, eight blood samples were taken from each patient without tourniquet in resting conditions for at least 30 minutes and without preceding exercise^44^. The first blood sample was obtained before breakfast at 7.30 am that corresponds to time point 1 (T1) after overnight fasting for 16 hours. The other 3 pre-prandial samples were obtained 30 minutes before lunch (T3), 4 pm snack (T5), and dinner (T7) at 11.30 am, 3.30 pm, and 7.30 pm, respectively. Four post-prandial samples were obtained 1 hour after meals (T2, T4, T6, T8). The different meals were standardised for all patients during hospital stay. Caloric values for every day breakfast and 4-pm snack were exactly the same; 358 Kcal and 135 Kcal, respectively. The mean caloric values of lunch and dinner over the 3-day hospitalisation were 885 Kcal and 446 Kcal, respectively for all patients. For lactate measurements, 2 ml of venous blood was collected in lithium heparin tubes and immediately transported to the Biochemistry Laboratory on ice. The plasma is then separated by centrifugation at 20° for 10 minutes X 2255 g. Quantitative assay of plasma lactate was measured with ADVIA^®^ Chemistry XPT system (Siemens Healthcare Diagnostics Ltd., UK) by an end point enzymatic reaction using lactate oxidase to oxidize lactate into pyruvate and hydrogen peroxide. Catalysed by peroxidase, the hydrogen peroxide formed reacts with a chromogen yielding a purple coloured substance. The resulting modification in adsorbance at 545/694 nm measured by spectrophotometry is proportional to the lactate concentration. References values for blood lactate were as follows: T1 (0.55–1.15 mmol/L), T2 (0.90–1.60 mmol/L), T3 (0.70–1.40 mmol/L), T4 (0.90–1.40 mmol/L), T5 (0.56–1.06 mmol/L), T6 (0.70–1.50 mmol/L), T7 (0.55–1.18 mmol/L), and T8 (0.73–1.45 mmol/L). A cut-off ≥2 mmol/L at any lactate time point defined elevated lactate levels. The lower limit of quantitation was 0.03 mmol/L, and the error measurement of the assay was 5.22%. Results of lactate cycle were unexploitable if more than 2 out of 8 time points measurements were missing, mainly due to haemolysis.

### Patients’ grouping

According to blood lactate concentrations, we stratified patients into two groups; the first one included patients in whom ≥1 lactate measurements were ≥2 mmol/L, and the other one comported patients with normal lactate concentrations.

### Statistical analysis

Categorical data were expressed as absolute number and percentage. Continuous data were expressed as median and interquartile (IQ). Data were compared using the Student t test for continuous variables and the χ² test or Fisher’s test for categorical data. Lactate analyses were compared using 2 way-ANOVA and lactate values were presented as means with 95% confidence interval in Fig. 2. Post-hoc analyses for each lactate time point were realised after 2-way ANOVA with Bonferroni corrected t-tests. The multivariate analysis for testing the association of comorbidities and the group of elevated lactates were carried out using binomial logistic regression. The continuous variable Age was transformed into a categorical variable with a cut-off equal to the median of the whole population (< or ≥34 years). Odds-ratio (OR) were calculated with their 95% confidence interval (CI). A p value of < 0.05 was considered significant. The analyses were performed using the Graphpad Prism v6.01 software (GraphPad Software, Inc., La Jolla, CA 92037 USA).

## Supplementary information

Lactate measurements data

## Data Availability

Data that support the findings of this study are available from the corresponding author upon reasonable request.

## References

[1] Prins JB, Meer JWvander, Bleijenberg G (2006). Chronic fatigue syndrome. The Lancet.

[2] Beyond Myalgic Encephalomyelitis/Chronic Fatigue Syndrome: Redefining an Illness. *Mil. Med*. **180**, 721–723 (2015). 10.7205/MILMED-D-15-00085 26126237

[3] Collin, S. M. & Crawley, E. Specialist treatment of chronic fatigue syndrome/ME: a cohort study among adult patients in England. *BMC Health Serv. Res*. **17** (2017). 10.1186/s12913-017-2437-3 PMC5513420 28709432

[4] Carruthers BM (2011). Myalgic encephalomyelitis: International Consensus Criteria: Review: ME: Intl. Consensus Criteria. J. Intern. Med..

[5] Fukuda K (1994). The chronic fatigue syndrome: a comprehensive approach to its definition and study. International Chronic Fatigue Syndrome Study Group. Ann. Intern. Med..

[6] Collin SM, Heron J, Nikolaus S, Knoop H, Crawley E (2018). Chronic fatigue syndrome (CFS/ME) symptom-based phenotypes and 1-year treatment outcomes in two clinical cohorts of adult patients in the UK and The Netherlands. J. Psychosom. Res..

[7] Jason LA (2010). Classification of Myalgic Encephalomyelitis/Chronic Fatigue Syndrome by Types of Fatigue. Behav. Med..

[8] DeLuca J, Johnson SK, Ellis SP, Natelson BH (1997). Sudden vs gradual onset of chronic fatigue syndrome differentiates individuals on cognitive and psychiatric measures. J. Psychiatr. Res..

[9] Kidd E (2016). The Relationship between Age and Illness Duration in Chronic Fatigue Syndrome. Diagnostics.

[10] Siegel SD (2006). Impaired natural immunity, cognitive dysfunction, and physical symptoms in patients with chronic fatigue syndrome: preliminary evidence for a subgroup?. J. Psychosom. Res..

[11] Moneghetti, K. J. *et al*. Value of Circulating Cytokine Profiling During Submaximal Exercise Testing in Myalgic Encephalomyelitis/Chronic Fatigue Syndrome. *Sci. Rep*. **8**, (2018). 10.1038/s41598-018-20941-w PMC5807550 29426834

[12] Kerr JR (2008). Gene Expression Subtypes in Patients with Chronic Fatigue Syndrome/Myalgic Encephalomyelitis. J. Infect. Dis..

[13] Filler K (2014). Association of mitochondrial dysfunction and fatigue: A review of the literature. BBA Clin..

[14] Plioplys AV, Plioplys S (1995). Serum Levels of Carnitine in Chronic Fatigue Syndrome: Clinical Correlates. Neuropsychobiology.

[15] Maes M (2009). Coenzyme Q10 deficiency in myalgic encephalomyelitis/chronic fatigue syndrome (ME/CFS) is related to fatigue, autonomic and neurocognitive symptoms and is another risk factor explaining the early mortality in ME/CFS due to cardiovascular disorder. Neuro Endocrinol. Lett..

[16] Shungu DC (2012). Increased ventricular lactate in chronic fatigue syndrome. III. Relationships to cortical glutathione and clinical symptoms implicate oxidative stress in disorder pathophysiology: VENTRICULAR LACTATE, OXIDATIVE STRESS AND CEREBRAL BLOOD FLOW IN CFS. NMR Biomed..

[17] Natelson BH (2017). Elevations of ventricular lactate levels occur in both chronic fatigue syndrome and fibromyalgia. Fatigue Biomed. Health Behav..

[18] Lengert N, Drossel B (2015). In silico analysis of exercise intolerance in myalgic encephalomyelitis/chronic fatigue syndrome. Biophys. Chem..

[19] Lane RJM, Burgess AP, Flint J, Riccio M, Archard LC (1995). Exercise responses and psychiatric disorder in chronic fatigue syndrome. BMJ.

[20] Kennedy G (2005). Oxidative stress levels are raised in chronic fatigue syndrome and are associated with clinical symptoms. Free Radic. Biol. Med..

[21] Fulle S (2000). Specific oxidative alterations in vastus lateralis muscle of patients with the diagnosis of chronic fatigue syndrome. Free Radic. Biol. Med..

[22] Richards RS, Roberts TK, McGregor NR, Dunstan RH, Butt HL (2000). Blood parameters indicative of oxidative stress are associated with symptom expression in chronic fatigue syndrome. Redox Rep..

[23] Bakken IJ (2014). Two age peaks in the incidence of chronic fatigue syndrome/myalgic encephalomyelitis: a population-based registry study from Norway 2008–2012. BMC Med..

[24] Chu, L., Valencia, I. J., Garvert, D. W. & Montoya, J. G. Onset Patterns and Course of Myalgic Encephalomyelitis/Chronic Fatigue Syndrome. *Front. Pediatr*. **7** (2019). 10.3389/fped.2019.00012 PMC6370741 30805319

[25] Salit IE (1997). Precipitating factors for the chronic fatigue syndrome. J. Psychiatr. Res..

[26] Vincent A (2012). Prevalence, Incidence, and Classification of Chronic Fatigue Syndrome in Olmsted County, Minnesota, as Estimated Using the Rochester Epidemiology Project. Mayo Clin. Proc..

[27] Aaron LA, Burke MM, Buchwald D (2000). Overlapping Conditions Among Patients With Chronic Fatigue Syndrome, Fibromyalgia, and Temporomandibular Disorder. Arch. Intern. Med..

[28] Castro-Marrero J (2017). Comorbidity in Chronic Fatigue Syndrome/Myalgic Encephalomyelitis: A Nationwide Population-Based Cohort Study. Psychosomatics.

[29] Van Den Eede F, Moorkens G, Van Houdenhove B, Cosyns P, Claes SJ (2007). Hypothalamic-Pituitary-Adrenal Axis Function in Chronic Fatigue Syndrome. Neuropsychobiology.

[30] Maes M, Mihaylova I, De Ruyter M (2006). Lower serum zinc in Chronic Fatigue Syndrome (CFS): Relationships to immune dysfunctions and relevance for the oxidative stress status in CFS. J. Affect. Disord..

[31] Berkovitz S, Ambler G, Jenkins M, Thurgood S (2009). Serum 25-hydroxy vitamin D levels in chronic fatigue syndrome: a retrospective survey. Int. J. Vitam. Nutr. Res. Int. Z. Vitam.- Ernahrungsforschung J. Int. Vitaminol. Nutr..

[32] Lane RJ (1998). Muscle fibre characteristics and lactate responses to exercise in chronic fatigue syndrome. J. Neurol. Neurosurg. Psychiatry.

[33] Lane RJM, Barrett MC, Taylor DJ, Kemp GJ, Lodi R (1998). Heterogeneity in chronic fatigue syndrome: evidence from magnetic resonance spectroscopy of muscle. Neuromuscul. Disord..

[34] Craig C (2015). Mitoprotective dietary approaches for Myalgic Encephalomyelitis/Chronic Fatigue Syndrome: Caloric restriction, fasting, and ketogenic diets. Med. Hypotheses.

[35] Michalsen A (2013). In-Patient Treatment of Fibromyalgia: A Controlled Nonrandomized Comparison of Conventional Medicine versus Integrative Medicine including Fasting Therapy. Evid. Based Complement. Alternat. Med..

[36] Wagner, D. *et al*. Psychometric properties of the CDC Symptom Inventory for assessment of Chronic Fatigue Syndrome. *Popul. Health Metr*. **3** (2005). 10.1186/1478-7954-3-8 PMC1183246 16042777

[37] Jason, L. *et al*. Fatigue Scales and Chronic Fatigue Syndrome: Issues of Sensitivity and Specificity. *Disabil. Stud. Q*. **31** (2011). PMC3181109 21966179

[38] So S, Evans M, Jason LA, Brown A (2015). Are Stamina and Fatigue Polar Opposites? A Case Study. J. Prev. Interv. Community.

[39] Stouten B (2005). Identification of ambiguities in the 1994 chronic fatigue syndrome research case definition and recommendations for resolution. BMC Health Serv. Res..

[40] Myalgic Encephalomyelitis/Chronic Fatigue Syndrome Standards - NINDS Common Data Elements. https://www.commondataelements.ninds.nih.gov/MECFS.aspx#tab=Data_Standards.

[41] Chalder T (1993). Development of a fatigue scale. J. Psychosom. Res..

[42] Krupp LB, LaRocca NG, Muir-Nash J, Steinberg AD (1989). The Fatigue Severity Scale: Application to Patients With Multiple Sclerosis and Systemic Lupus Erythematosus. Arch. Neurol..

[43] Larson RD (2013). Psychometric Properties of the Modified Fatigue Impact Scale. Int. J. MS Care.

[44] Parikh S (2015). Diagnosis and management of mitochondrial disease: a consensus statement from the Mitochondrial Medicine Society. Genet. Med..

