# Supplementary material for: Elevated blood lactate in resting conditions correlate with post-exertional malaise severity in patients with Myalgic encephalomyelitis/Chronic fatigue syndrome
Source: Sci Rep. 2019 Dec 11;9:18817. doi: 10.1038/s41598-019-55473-4 (PMC6906377; doi:10.1038/s41598-019-55473-4)
Supplement: Supplementary file 1 — Lactate measurements data [file 41598_2019_55473_MOESM1_ESM.pdf]

Elevated blood lactate in resting conditions correlate with post-exertional malaise severity in patients with Myalgic encephalomyelitis/Chronic fatigue syndrome

Alaa Ghali, Carole Lacout, Maria Ghali, Aline Gury, Anne-Berengere Beucher, Pierre Lozac'h, Christian Lavigne, Geoffrey Urbanski.

Supplementary Table SI 1 Lactate measurements data

ID	Elevated lactates	T1	T2	T3	T4	T5	T6	T7	T8
1	1	0,59	2,06	0,7	1,67	1,07	1,33	1,04	1,37
2	1	NA	2,47	1,43	1,76	1,59	1,29	1,06	1,83
6	1	1,35	2,12	1,35	1,35	1,23	1,95	1,76	NA
8	1	0,6	2,44	1,38	1,63	1,05	1,57	1,45	1,3
19	1	0,79	2,34	NA	1,54	0,89	1,76	1,3	1,35
20	1	0,79	2,01	0,93	1,41	0,93	1,26	0,81	1,94
23	1	NA	2,11	1,26	1,02	1,17	1,24	1,94	0,86
27	1	0,69	2,06	1,22	1,4	0,99	1,22	1,29	2,02
41	1	1,53	1,68	1,19	1,78	1,83	1,59	2,12	1,93
43	1	0,69	1,27	0,97	1,3	1,2	1,28	2,15	1,2
44	1	2,32	0,82	0,88	0,68	1,35	1,26	1,23	0,82
46	1	NA	2,24	1,14	0,8	0,89	1,1	1,25	1,1
55	1	1,43	2,76	1,64	1,21	1,05	1,4	1,1	1,94
58	1	1,01	1,71	0,93	1,63	1,15	1,34	1,03	2,54
62	1	1,27	2,96	2,02	NA	1,43	1,42	1,17	1,97
72	1	0,92	1,38	1,65	1,37	1,11	1,11	0,89	2,21
74	1	0,6	1,92	1,98	1,14	0,92	1,15	NA	2,25
78	1	0,91	1,97	1,11	1,31	1,13	0,92	0,79	2,08
79	1	0,81	2,04	0,83	1,62	1	1,39	0,87	0,81
82	1	0,92	2,31	1,49	1,83	1,3	1,4	0,98	1,88
84	1	1,08	2,62	0,79	1,39	1,31	1,53	0,93	2,33
86	1	1,03	2,79	1,36	1,71	1,32	1,41	1,15	2,32
87	1	0,77	2,15	1,17	1,46	1,4	1,45	NA	1,14
90	1	0,79	2,12	0,77	0,57	0,7	0,89	0,61	NA
94	1	2,53	NA	1,92	2,32	1,94	1,8	1,75	1,96
103	1	1,43	2,15	1,5	2,25	1,44	1,4	NA	1,72
105	1	0,95	2,41	1,65	1,84	1,52	NA	1,61	1,55
108	1	1,1	2,16	0,98	1,79	1,46	NA	1,12	1,9
109	1	1,25	2,41	1,16	1,74	1	2,45	0,55	1,84

110	1	0,52	2,1	0,66	1,57	NA	0,91	0,79	1,46
112	1	1,18	3,02	0,93	2,22	1,77	1,16	0,78	1,63
115	1	1,26	2,17	1,25	1,84	1,64	1,6	1,07	2,79
119	1	0,71	1,58	1,17	2,56	1,05	1,07	0,9	1,41
122	1	4,06	3,84	2,58	2,96	2,18	2,55	2,54	3,14
123	1	0,99	1,25	NA	1,27	NA	2,37	0,57	1,65
128	1	1,66	1,93	1,78	1,23	2,14	1,15	0,77	1,51
129	1	0,82	1,88	0,8	2,18	2,06	1,4	0,82	NA
131	1	0,51	1,98	1,24	2,15	1,54	1,03	1,05	1,4
135	1	1,22	2,18	1,02	1,5	1,25	1,54	0,89	2
136	1	0,8	0,8	2,41	1,17	NA	1,23	1,14	NA
137	1	1,07	2,62	NA	2,29	1,57	1,69	1,59	1,96
139	1	0,7	2,07	1,85	NA	NA	1,06	0,67	1,5
143	1	NA	2,82	1,25	1,84	NA	1,57	1,27	2,08
144	1	0,73	2,35	0,95	1,67	1,06	NA	1,33	1,39
146	1	NA	2,39	1,32	1,82	1,36	1,17	0,8	2,1
147	1	1,57	2,47	1,74	2,05	1,46	1,43	1,16	2,22
148	1	1,07	2,14	1,13	1,2	1,1	1,4	1,17	1,42
149	1	0,75	2,59	1,2	0,86	0,67	2,13	0,74	1,13
151	1	0,82	2,88	1,93	1,56	1,53	1,47	1,9	1,68
152	1	0,81	2,35	0,56	1,19	0,61	0,56	0,5	0,93
159	1	0,76	2,16	0,64	0,86	0,82	0,73	0,47	1,6
160	1	1,84	2,58	1,29	1,66	0,99	1,76	1,15	1,95
161	1	0,71	2,62	NA	1,67	1,53	2,01	1,31	1,23
163	1	1,2	2,26	1,39	1,52	NA	1,94	1,05	1,82
167	1	0,96	2,35	1,03	1,21	NA	1,88	1,07	1,59
7	0	NA	1,5	0,78	NA	1,85	0,9	0,63	1,81
10	0	0,63	1,68	1,25	1,49	0,85	0,61	0,89	1,15
12	0	0,58	1,24	1,04	1,48	1,1	1,27	1,26	1,37
16	0	0,64	1,7	1,07	1,49	1,3	1,22	0,62	1,49
18	0	0,79	1,66	0,94	1,62	1,36	1,59	0,81	1,51
21	0	0,57	1,1	1,3	1,55	0,92	1,11	0,92	1,1
22	0	0,52	NA	0,9	1,06	NA	1,17	0,99	0,7
26	0	1,31	1,16	0,76	1,16	0,92	1,02	0,76	1,49
30	0	1,44	1,77	0,86	1,27	0,91	1,19	1,18	1,52
32	0	0,57	1,92	1,76	1,71	1,23	1,29	0,87	1,38
33	0	0,55	0,84	0,95	0,55	0,56	0,59	0,43	1,08
35	0	0,49	1,82	1,29	1,42	1,26	0,87	0,97	1,48

36	0	0,6	1,17	0,69	0,95	0,68	NA	0,79	0,6
37	0	0,55	1,39	0,99	1,16	0,97	1,23	0,87	1,48
38	0	0,57	1,35	0,67	1,59	0,93	0,72	0,75	1,54
39	0	NA	1,9	0,73	1,31	0,97	1,03	1,14	1,16
40	0	0,53	1,69	NA	1,29	1,07	1,1	1,06	1,45
42	0	0,52	0,87	0,39	0,99	0,52	1,11	NA	1,37
48	0	0,49	1,01	0,5	0,99	0,84	1,04	1,04	1,17
49	0	0,66	1,46	1,37	1,52	1,17	0,81	1,12	1,55
50	0	0,69	1,03	0,66	1,07	0,62	1,19	0,5	1,08
52	0	1,31	1,94	1,7	1,37	1,38	NA	NA	1,54
56	0	0,61	NA	0,92	0,92	1,4	1,2	0,75	0,91
57	0	0,66	1,54	1,12	0,85	1,18	0,96	1,21	1,06
59	0	0,65	NA	1,75	1,21	1,03	1,88	1,37	1,93
60	0	0,51	1,67	0,97	1,29	0,63	1,1	1	1,31
64	0	0,79	1,11	0,75	0,89	0,57	0,64	0,59	0,73
65	0	0,66	NA	0,54	1,56	1,29	1,31	0,79	1,66
66	0	0,48	1,79	0,66	1,33	1,03	0,85	0,77	1,38
69	0	0,63	1,72	1,24	1,4	0,87	1,05	NA	1,78
71	0	0,82	1,61	1,68	1,51	1,27	1,72	NA	1,58
76	0	1	1,51	0,81	1,89	0,89	0,87	0,83	1,37
80	0	0,55	1,01	NA	0,9	0,62	0,82	0,6	0,9
81	0	0,82	1,36	1,09	1,01	0,83	0,63	0,65	NA
83	0	0,89	1,17	1,12	0,96	1,08	1,01	0,78	1,23
88	0	0,81	1,45	0,57	0,9	0,76	0,94	0,86	1,11
89	0	0,6	1,03	0,92	0,58	0,52	0,97	0,59	0,48
91	0	0,48	1,65	0,79	1,22	0,98	NA	0,99	1,45
92	0	0,57	1,38	0,59	NA	1,34	1,06	0,73	1,68
95	0	1,72	1,76	0,87	1,32	1,09	1,16	1,11	1,26
96	0	1,56	1,7	1,24	1,54	1,08	1,31	1,09	1,38
99	0	0,93	1,35	1,34	1,55	1,3	1,15	0,78	1,78
100	0	0,79	1,5	0,81	1,15	0,81	1,23	0,9	0,91
101	0	0,91	1,8	0,78	1,39	0,67	1,94	0,64	NA
102	0	1,11	1,5	0,78	1,67	0,89	0,55	0,66	1,21
104	0	0,74	1,76	0,94	1,41	NA	NA	0,78	1,1
106	0	0,79	1,2	0,88	1,33	0,97	1,25	0,9	NA
111	0	1,15	1,82	0,97	1,98	1,46	1,26	0,98	1,75
117	0	0,83	1,48	0,97	1,26	1,36	1,54	0,89	1,56

118	0	0,84	1,94	1,41	1,43	1,13	1,54	1,5	1,81
120	0	0,96	1,92	1,64	1,29	1,25	1,46	0,87	1,37
121	0	1,96	1,95	NA	1,57	1,3	1,56	0,71	1,93
125	0	1,01	1,73	1,16	1,43	0,99	1,11	1,07	1,21
130	0	0,79	NA	0,92	1,67	1,13	0,72	0,52	1,12
132	0	0,62	0,98	0,65	0,83	0,92	1,43	0,91	1,53
133	0	0,63	1,78	1,52	1,14	0,95	1,52	1,44	0,85
138	0	0,39	1,17	1,24	0,67	0,44	0,75	0,36	0,74
153	0	0,96	NA	0,84	1,2	1,03	1,07	1,21	1,01
154	0	0,66	1,51	1	1,23	NA	1,23	1,29	1,37
155	0	0,56	1,12	1,28	1,25	1,21	1,25	1,2	1,7
156	0	0,71	1,62	1,26	1,32	1,02	1,17	1,46	1,9
157	0	0,43	1,19	0,84	1,11	0,82	0,53	0,53	1,46
158	0	0,46	1,47	0,83	1,61	1	0,83	0,96	1,35
162	0	0,71	1,88	1,48	1,38	1,6	1,11	0,98	1,56
164	0	0,59	0,58	0,86	0,88	0,65	0,77	0,72	1,34
165	0	0,56	NA	0,83	1,21	0,62	1,74	0,98	0,97
166	0	1,26	1,6	1,01	1,34	1,31	NA	1,14	NA
168	0	0,67	1,86	0,94	0,96	0,74	1,27	0,75	NA

Elevated blood lactate levels are equal or more than 2 mmol/L
